# Steroids for the Treatment of Misophonia and Misokinesia

**DOI:** 10.1155/2024/3976837

**Published:** 2024-01-16

**Authors:** Jadon Webb, Afton Williamson

**Affiliations:** ^1^Bloom Mental Health, LLC, 26 W Dry Creek Circle, Suite 710, Littleton, CO 80120, USA; ^2^Child Study Center, Yale School of Medicine, New Haven, USA

## Abstract

Misophonia and misokinesia are disorders characterized by intensely negative physical and emotional reactions to specific auditory and visual stimuli. The availability of effective treatments, especially pharmacological ones, is limited. This report presents a case of a 35-year-old male with severe misophonia and misokinesia who experienced nearly complete resolution of symptoms while undergoing high-dose steroid therapy for an unrelated muscular injury. Two days after starting a 20 mg oral prednisone taper pack (in which the steroid dose is reduced by 4 mg daily), his Amsterdam Misophonia Scale (A-Miso-S) score drastically reduced from a baseline of 23 (i.e., extreme symptoms) to 1, with symptom relief persisting for approximately 2 weeks after completing the taper. Months later, a daily dose of prednisone (4 mg) was reintroduced. This again resulted in a marked reduction in symptoms (A-Miso-S of 6), enabling him to resume working in an office setting despite his triggers. Symptom improvement remained stable over several months. This case raises the possibility of the steroid prednisone as a novel treatment for misophonia and misokinesia. However, further investigation is needed to determine the generalizability of this observation.

## 1. Introduction

Misophonia is a condition characterized by marked emotional and physiological aversion to specific sounds. Misokinesia is a similar response to certain visual stimuli. These conditions are only recently recognized [1] and are becoming a topic of increasing clinical interest, as they appear to be quite common and can be quite distressing [2].

Consensus work groups are refining the diagnostic criteria for misophonia and misokinesia [3]. The emotional and autonomic symptoms experienced in response to triggers overlap those observed in anxiety-spectrum psychiatric disorders such as phobias, panic disorder, and posttraumatic stress disorder (PTSD). However, misophonia and misokinesia are not currently recognized in diagnostic manuals such as the International Classification of Diseases, 11th Revision (ICD-11) [4], or the Diagnostic and Statistical Manual of Mental Disorders, 5th Edition, Text Revision (DSM-5-TR) [5], reflecting the need for a deeper understanding of their etiology, and further standardization of diagnostic criteria.

Thus far, no randomized trials have assessed the effects of any medication on misophonia or misokinesia symptoms. Published reports suggest that antidepressants [6, 7], beta-blockers such as propranolol [8], and mood-stabilizing antipsychotics such as risperidone [9, 10] may alleviate symptoms in some cases. Potent psychotomimetic drugs, such as psilocybin, ketamine, and MDMA, hold theoretical potential for addressing some of the core symptoms of misophonia and misokinesia, although this is still a hypothesis that will require clinical assessment [11].

This report contributes to the existing literature by documenting a case in which prednisone, a synthetic corticosteroid, was highly effective in treating severe misophonia and misokinesia. This case suggests a potential role for corticosteroids in managing this complex problem that otherwise still has few treatment options. It also raises questions regarding the underlying pathophysiology and the potential in general for anti-inflammatory approaches.

## 2. Case Presentation

### 2.1. Case Summary

The patient, a 35-year-old white male, sought evaluation and treatment for misophonia and misokinesia symptoms. He was in considerable distress and had taken medical leave from his office job due to severe triggers incited by everyday sounds and actions from coworkers. His primary treatment objective was to mitigate these trigger responses to manageable levels, thereby allowing his return to work.

### 2.2. Misophonia History

The patient first experienced misophonia symptoms at the age of 12, and the severity of his trigger reactions slowly increased over the years. Triggers included chewing food or gum, crunching foods, lip smacking, repetitive tapping, and seeing someone shake their leg, all of which were common in his office job.

The patient's trigger reactions were fairly typical for misophonia/misokinesia. His primary emotional response was an intense sensation of anger or rage. This frequently resulted in an exaggerated focus on the trigger, characterized by a desire to confront the trigger source to compel it to stop, although he never exhibited aggressive behavior. Feelings of anxiety and panic were also common, particularly when he felt trapped in a triggering situation. Once conscious of a trigger, his thoughts became consumed by it, and he was unable to shift focus until the trigger ceased, and he would additionally feel an overwhelming compulsion to exit the situation. In terms of physical response, triggers led to immediate muscle tension and clenching of his fists. Following a trigger event, he would feel exhausted and depressed.

### 2.3. Impairment and Distress from Triggers

When the patient first sought our assistance, his misophonia severity, as measured by the Amsterdam Misophonia Scale (A-Miso-S) [12], was 23, indicative of “extreme” symptoms. Trigger responses to family and coworkers caused significant life impairment, including having to take medical leave from his job and being increasingly unable to tolerate being physically near to his father, with whom he otherwise shared a good relationship. This impairment was associated with a growing sense of hopelessness that things could ever improve and passive suicidal thoughts.

### 2.4. Medical and Psychiatric History

The patient is an intelligent, articulate 35-year-old engineer who takes great pride in his highly technical work. He has a decades-long history of major depression, treated periodically with the antidepressant escitalopram 20 mg daily, which was partially effective for depression but did not affect misophonia/misokinesia symptoms. He did not consume nicotine, alcohol, or any illicit substances and had no other known psychiatric disorders.

### 2.5. Previous Attempts at Misophonia Treatment

Auditory interventions such as earplugs and sound-generating devices were minimally effective, and he had not previously attempted any behavioral or psychotherapeutic interventions. Adequate sleep, aerobic exercise, and a healthy diet all modestly alleviated the intensity of triggers; however, these self-care measures were insufficient to allow him to continue to work and socialize normally with others.

### 2.6. Treatment Using the Steroid Prednisone

**Figure 1:**
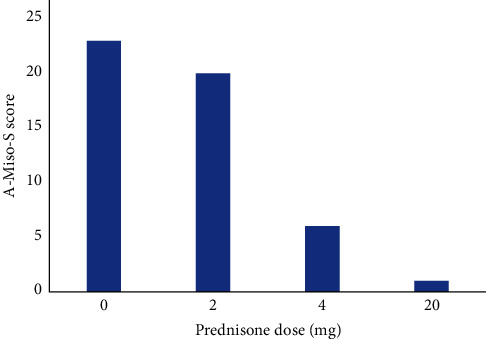
Prednisone dose vs. A-Miso-S score. The severity of misophonia symptoms experienced by this patient, as measured by the A-Miso-S rating scale vs. the dose of prednisone. Lower scores mean less misophonia symptoms. Of note, 0 mg was his score when not taking steroids, 2 and 4 mg were daily oral doses of prednisone, and 20 mg was the starting dose of a 5-day prednisone taper pack in which the dose was then decreased by 4 mg daily, the score shown here was from his first week of treatment on the steroid pack.

A medical provider from another facility initiated a high-dose prednisone tapering regimen to treat a sports-related abdominal muscle tear. The initial dose was prednisone 20 mg on Day 1, reducing by 4 mg each day until tapering off in 5 days. Within 2 days of starting the prednisone taper, he experienced a remarkable improvement in his misophonia and misokinesia symptoms. His A-Miso-S score dropped sharply from 23 to 1, indicating near-complete symptom relief (see Figure 1 for a comparison of A-Miso-S scores with prednisone dosage).

Subjectively, the patient reported that upon taking the steroid pack, “for the first time in a long time…I felt normal,” meaning that his misophonia/misokinesia symptoms were not severely affecting his daily life. His overall mood moderately improved during the steroid taper, but importantly, we did not identify any signs of mania, a potential steroid side effect. His sleep was unaffected, and there were no symptoms of euphoria, excessive energy, rushed speech, grandiosity, or other unusual changes. The substantial reduction in misophonia/misokinesia symptoms well exceeded what we would have expected from his modest change in mood.

Two weeks after completing the prednisone taper, the patient's misophonia symptoms slowly returned to their original severity. Given his previous steroid response, but with concern about long-term risks from the use of steroids, we initially attempted a low dose (2 mg) daily prednisone regimen. This resulted in minimal improvement (A-Miso-S score of 20, see Figure 1), which we and the patient both deemed unsatisfactory. We thus increased the prednisone dose to 4 mg daily, which dramatically improved the misophonia/misokinesia symptoms (A-Miso-S score of 6, or mild symptoms).

While taking 4 mg daily prednisone, the patient was still aware of triggers at work but experienced little emotional distress from them, stating, “my fuse is so much longer… it would have taken much more triggering exposure to feel angry.” He also reported minimal physical symptoms from triggers and overall did not feel impaired when they occurred. He was thus able to return to work in the office with no impairment, which he found profoundly helpful. He also reconnected with his father, now able to tolerate his triggers even for extended periods of time together. He reported high overall satisfaction with the treatment, with a total resolution of hopeless and passive suicidal thoughts. His affect on recent visits to our clinic was bright, engaged, pleasant, and hopeful about the future.

Symptom improvement was sustained for several months of treatment up to the point of writing this report. No side effects from the steroids were reported or noted on the exam. Going forward, the treatment plan was regular monitoring for side effects while exploring alternate potential treatments that may have fewer long-term side effects and/or may allow him to periodically take steroid holidays.

## 3. Discussion

The use of oral prednisone, in this case, elicited a clearly observable, replicable, stable, and dose-dependent clinical response. This remarkable response raises intriguing possibilities for developing new medication treatments and perhaps better understanding the mechanism underlying this illness.

That said, we note that other misophonia/misokinesia patients in our clinic who also incidentally took steroids have *not* experienced similarly clear symptom relief. Thus, it already seems clear that prednisone is not a universally effective treatment for misophonia/misokinesia, and this one case should not be interpreted as generalized treatment guidance. The side effects of steroids are a major consideration likely to limit this option, particularly for milder cases in which the risks of treatment are more likely to outweigh the benefits. Even in this extreme case, we are likely to continue to explore alternative medications to limit long-term side effects.

### 3.1. Possible Mechanism of Steroid Action on Misophonia and Misokinesia

The mechanism through which steroids may alleviate misophonia and misokinesia symptoms is unknown. Steroids can modify auditory processing at the cortical level, as shown in animal models given exogenous steroids [13]. Steroids can also affect psychiatric disorders characterized by abnormal emotional and physical responses to generally benign stimuli, such as PTSD. Steroid treatment may improve symptoms and facilitate fear extinction during PTSD treatment, particularly in patients who show high physiological responsiveness to a test dose of steroid [14, 15].

Inflammation is increasingly thought to underlie many psychiatric conditions [16] and is increased during high stress and sleep deprivation. Misophonia symptoms are frequently reported as more intense after poor sleep and during stress. Psychiatric medications, including selective serotonin reuptake inhibitor antidepressants, may function in part by modulating inflammation [17], and a wide range of anti-inflammatory medications may be effective in treating psychiatric conditions [18]. If inflammation is a key driver in some cases of misophonia/misokinesia, perhaps steroids work by counteracting this. If so, then other anti-inflammatory agents may also be of interest to consider, including nonsteroidal anti-inflammatory drugs, polyunsaturated fatty acids, cytokine modulators, and atypical anti-inflammatories such as statins and minocycline.

### 3.2. Future Directions

Future investigations should systematically analyze cases of steroid-induced misophonia/misokinesia symptom relief to ascertain if this treatment has wider applicability or is a rare occurrence. This could help guide whether it is worthwhile to pursue controlled clinical trials. Identifying potential subpopulations likely to be steroid-responsive may also be beneficial, perhaps by measuring biomarkers of inflammation and assessing a patient's innate responsiveness to a steroid challenge, such as through the dexamethasone suppression test.

### 3.3. Summary

The clear, positive response of misophonia and misokinesia symptoms to the oral steroid prednisone reported in this case expands potential treatment options for a problem that presently has no established medical treatment. It also clearly invites further exploration of the generalizability of this finding.

## Data Availability

No datasets to share.
